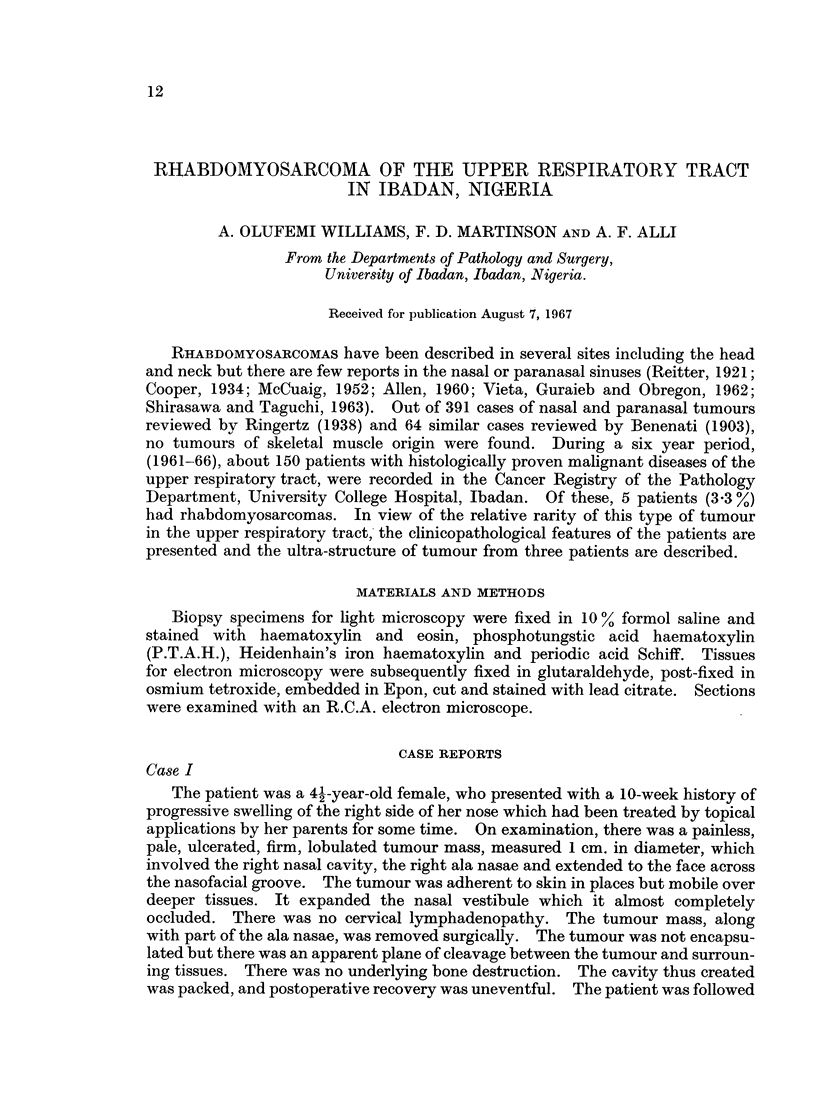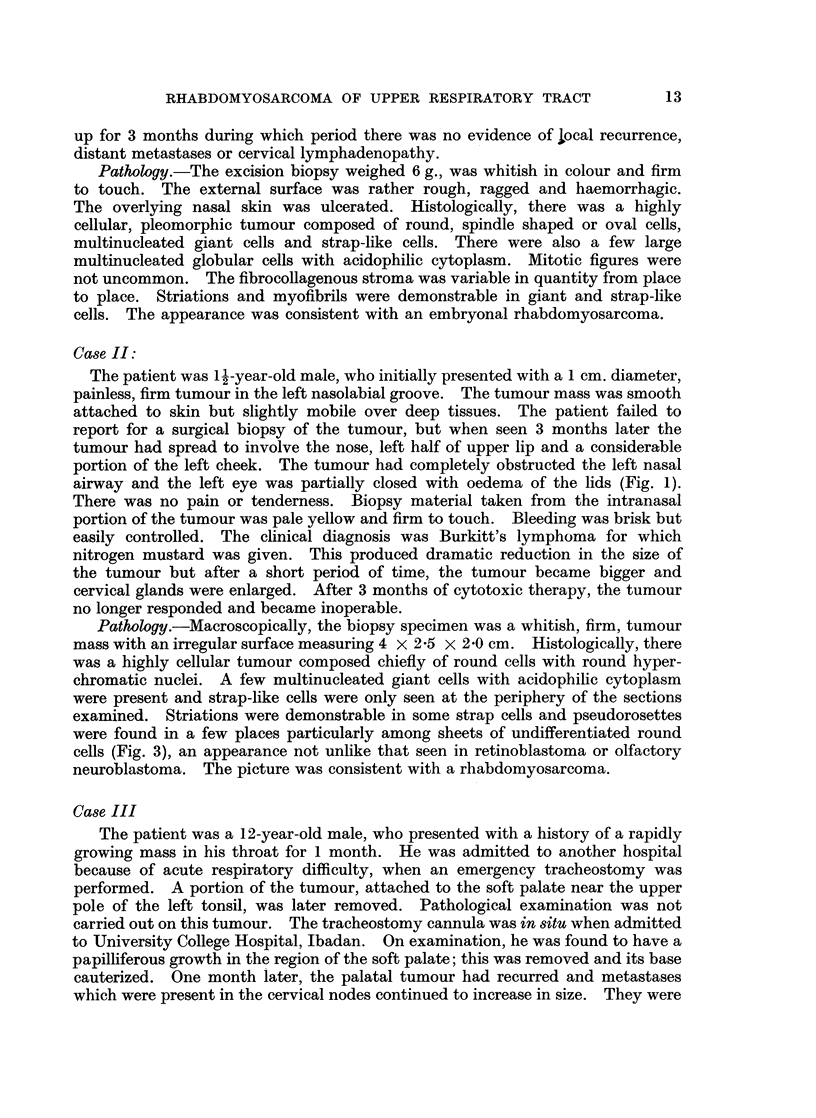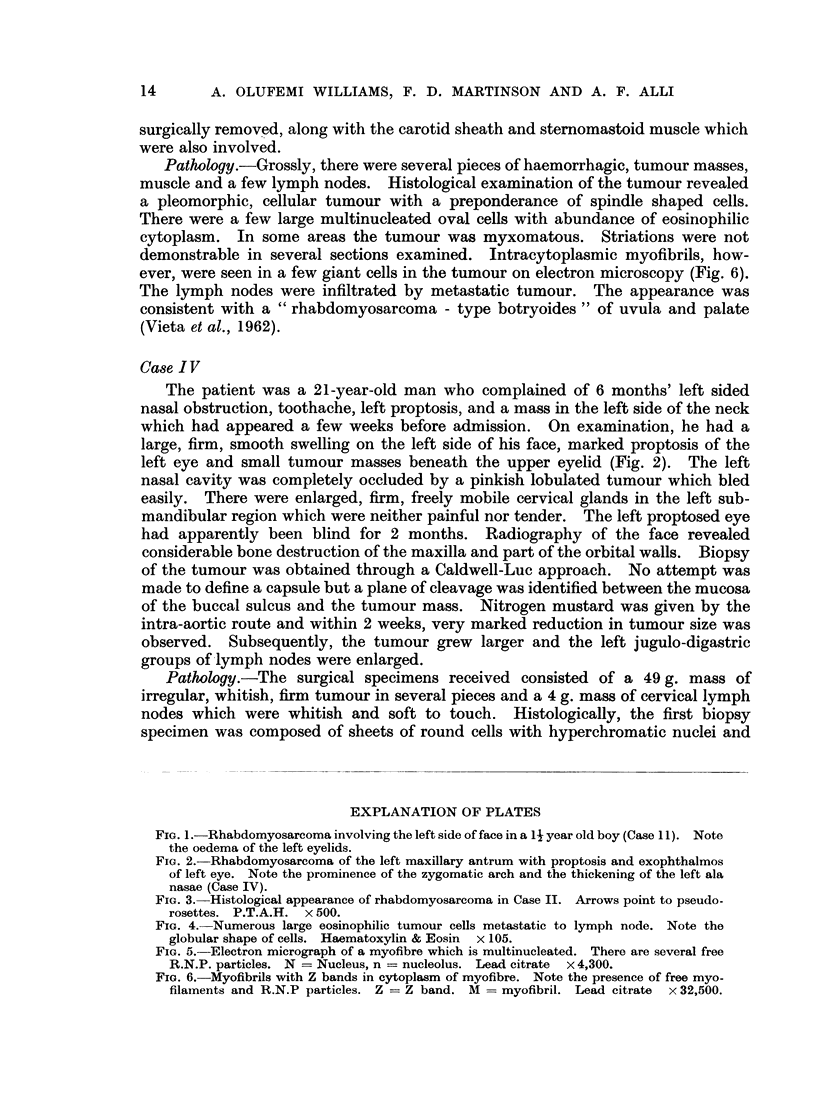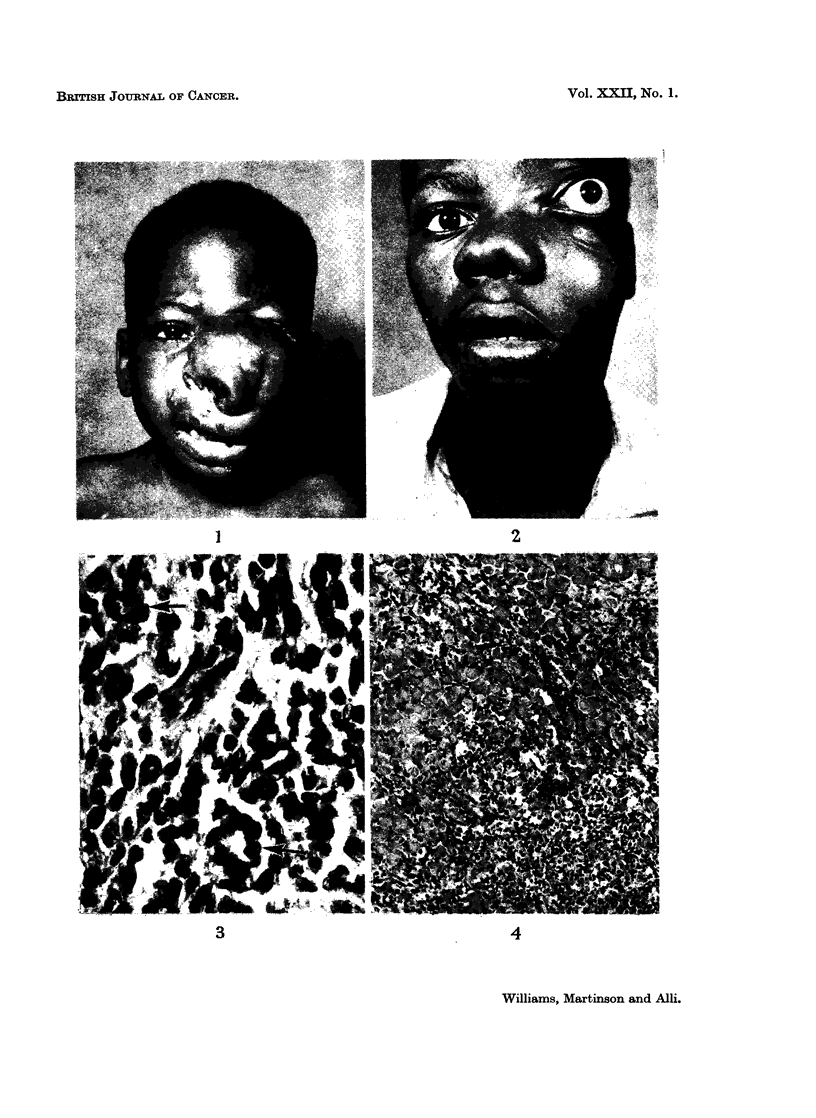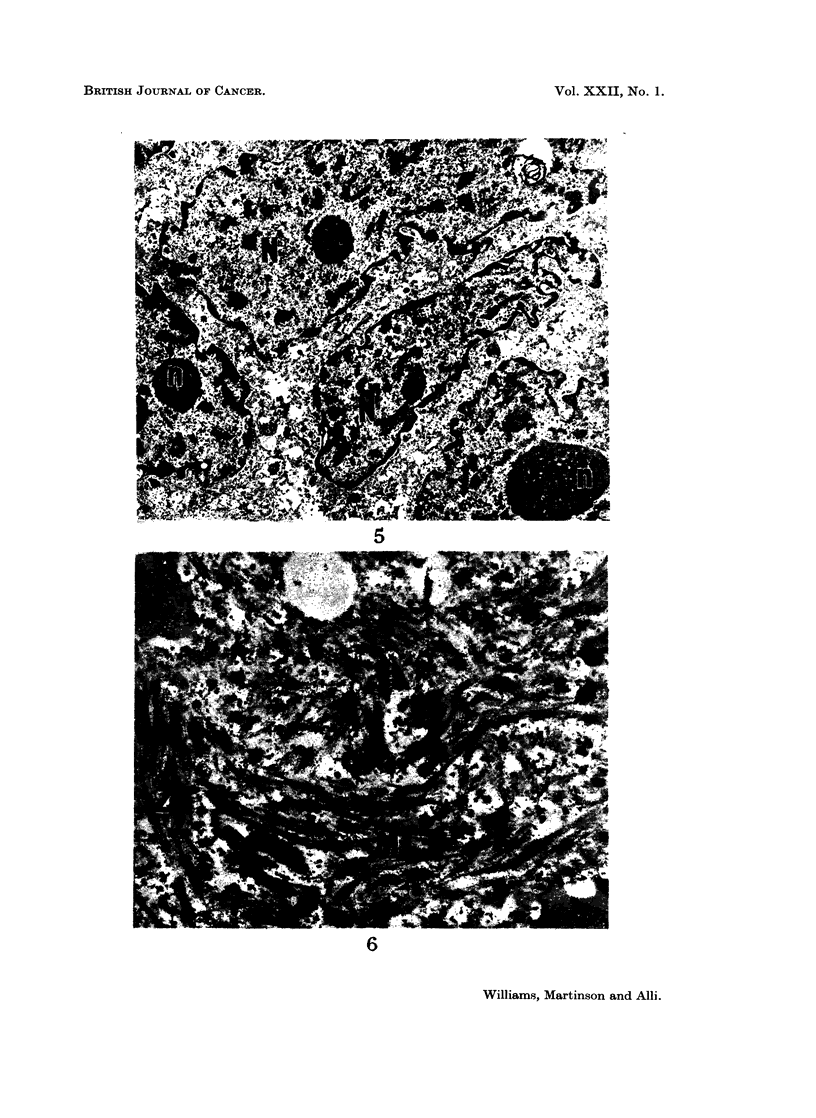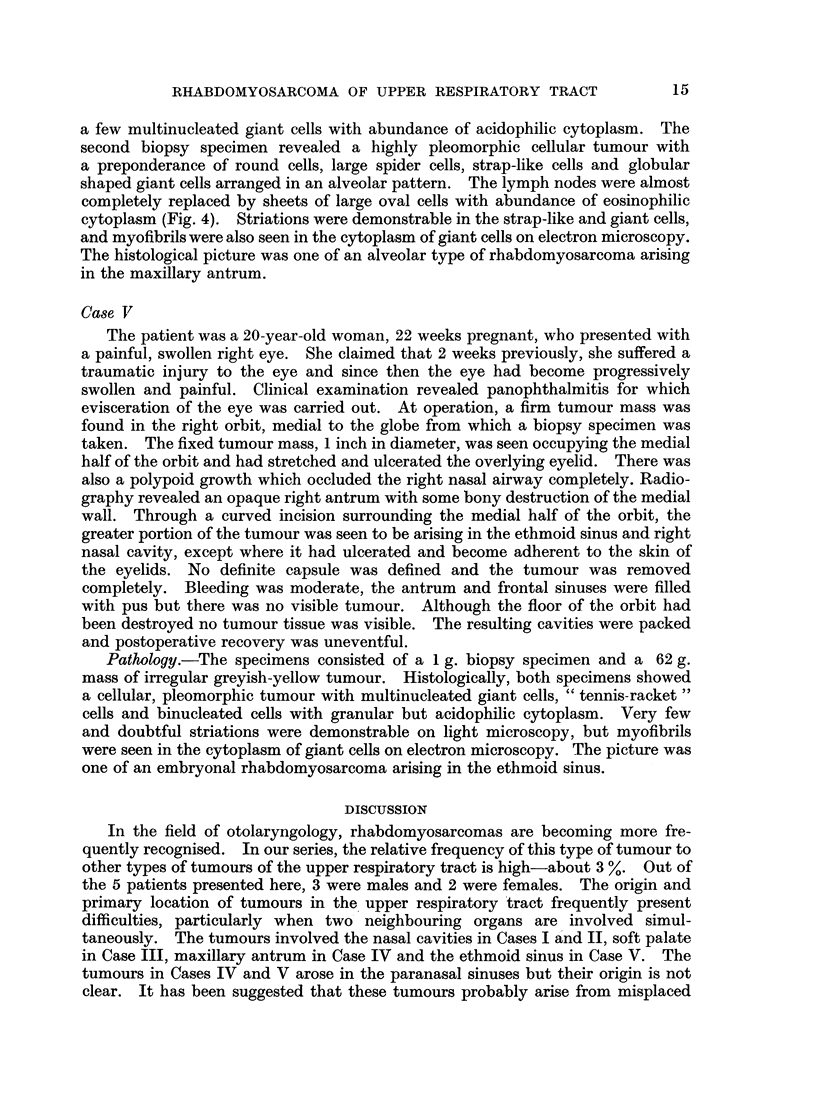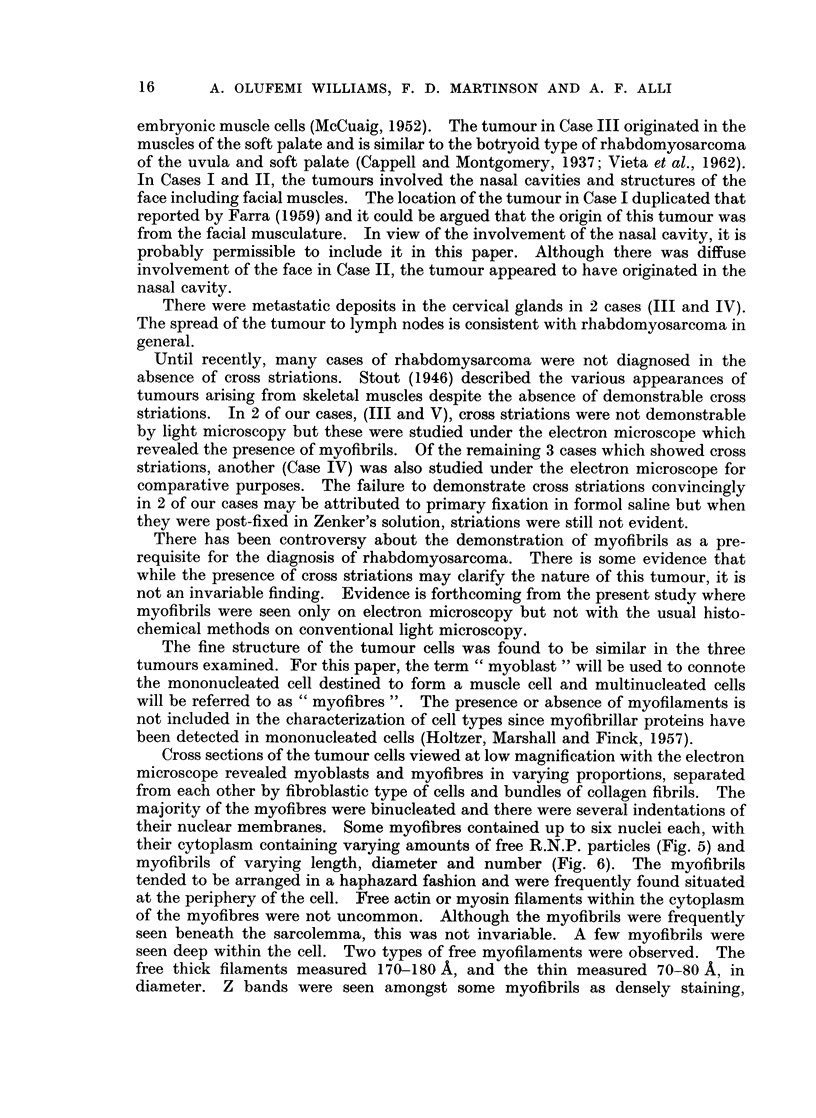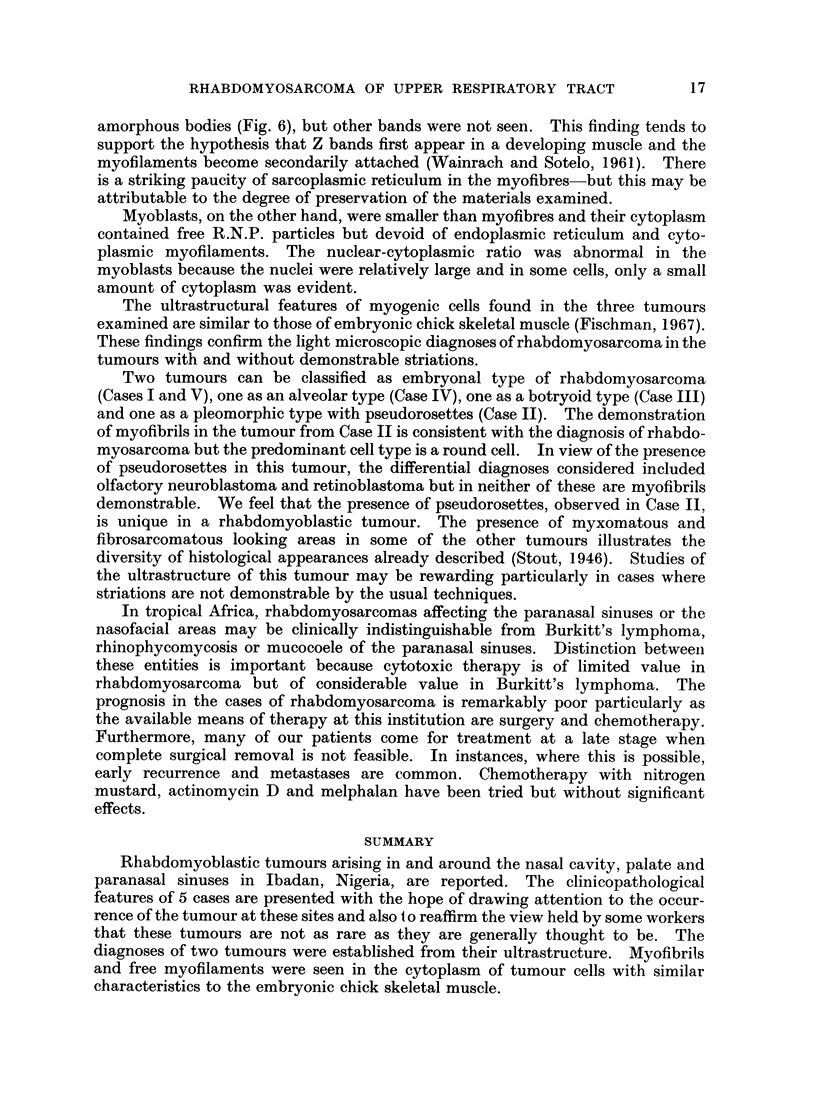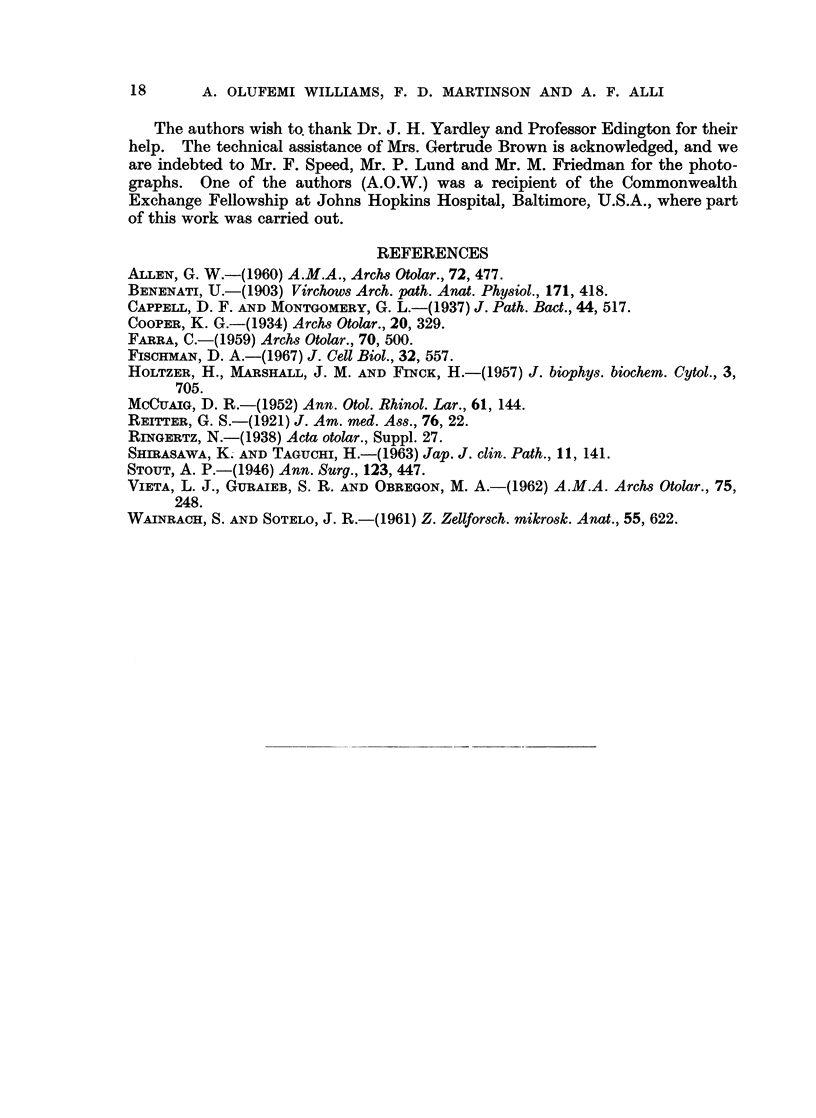# Rhabdomyosarcoma of the upper respiratory tract in Ibadan, Nigeria.

**DOI:** 10.1038/bjc.1968.3

**Published:** 1968-03

**Authors:** A. O. Williams, F. D. Martinson, A. F. Alli

## Abstract

**Images:**


### RHABDOMYOSARCOMA OF THE UPPER RESPIRATORY TRACT IN IBADAN, NIGERIA

A. OLUFEMI WILLIAMS, F. D. MARTINSON AND A. F. ALLI

*From the Departments of Pathology and Surgery, University of Ibadan, Ibadan, Nigeria.*

Received for publication August 7, 1967

RHABDOMYOSARCOMAS have been described in several sites including the head and neck but there are few reports in the nasal or paranasal sinuses (Reitter, 1921; Cooper, 1934; McCuaig, 1952; Allen, 1960; Vieta, Guraieb and Obregon, 1962; Shirasawa and Taguchi, 1963). Out of 391 cases of nasal and paranasal tumours reviewed by Ringertz (1938) and 64 similar cases reviewed by Benenati (1903), no tumours of skeletal muscle origin were found. During a six year period, (1961–66), about 150 patients with histologically proven malignant diseases of the upper respiratory tract, were recorded in the Cancer Registry of the Pathology Department, University College Hospital, Ibadan. Of these, 5 patients (3·3 %) had rhabdomyosarcomas. In view of the relative rarity of this type of tumour in the upper respiratory tract, the clinicopathological features of the patients are presented and the ultra-structure of tumour from three patients are described.

### MATERIALS AND METHODS

Biopsy specimens for light microscopy were fixed in 10 % formol saline and stained with haematoxylin and eosin, phosphotungstic acid haematoxylin (P.T.A.H.), Heidenhain's iron haematoxylin and periodic acid Schiff. Tissues for electron microscopy were subsequently fixed in glutaraldehyde, post-fixed in osmium tetroxide, embedded in Epon, cut and stained with lead citrate. Sections were examined with an R.C.A. electron microscope.

### CASE REPORTS

### *Case I*

The patient was a 4½-year-old female, who presented with a 10-week history of progressive swelling of the right side of her nose which had been treated by topical applications by her parents for some time. On examination, there was a painless, pale, ulcerated, firm, lobulated tumour mass, measured 1 cm. in diameter, which involved the right nasal cavity, the right ala nasae and extended to the face across the nasofacial groove. The tumour was adherent to skin in places but mobile over deeper tissues. It expanded the nasal vestibule which it almost completely occluded. There was no cervical lymphadenopathy. The tumour mass, along with part of the ala nasae, was removed surgically. The tumour was not encapsulated but there was an apparent plane of cleavage between the tumour and surrounding tissues. There was no underlying bone destruction. The cavity thus created was packed, and postoperative recovery was uneventful. The patient was followed up for 3 months during which period there was no evidence of local recurrence, distant metastases or cervical lymphadenopathy.

*Pathology.*—The excision biopsy weighed 6 g., was whitish in colour and firm to touch. The external surface was rather rough, ragged and haemorrhagic. The overlying nasal skin was ulcerated. Histologically, there was a highly cellular, pleomorphic tumour composed of round, spindle shaped or oval cells, multinucleated giant cells and strap-like cells. There were also a few large multinucleated globular cells with acidophilic cytoplasm. Mitotic figures were not uncommon. The fibrocollagenous stroma was variable in quantity from place to place. Striations and myofibrils were demonstrable in giant and strap-like cells. The appearance was consistent with an embryonal rhabdomyosarcoma.

*Case II:*

The patient was 1½-year-old male, who initially presented with a 1 cm. diameter, painless, firm tumour in the left nasolabial groove. The tumour mass was smooth attached to skin but slightly mobile over deep tissues. The patient failed to report for a surgical biopsy of the tumour, but when seen 3 months later the tumour had spread to involve the nose, left half of upper lip and a considerable portion of the left cheek. The tumour had completely obstructed the left nasal airway and the left eye was partially closed with oedema of the lids (Fig. 1). There was no pain or tenderness. Biopsy material taken from the intranasal portion of the tumour was pale yellow and firm to touch. Bleeding was brisk but easily controlled. The clinical diagnosis was Burkitt's lymphoma for which nitrogen mustard was given. This produced dramatic reduction in the size of the tumour but after a short period of time, the tumour became bigger and cervical glands were enlarged. After 3 months of cytotoxic therapy, the tumour no longer responded and became inoperable.

*Pathology.*—Macroscopically, the biopsy specimen was a whitish, firm, tumour mass with an irregular surface measuring $4 \times 2{\cdot}5 \times 2{\cdot}0$ cm. Histologically, there was a highly cellular tumour composed chiefly of round cells with round hyperchromatic nuclei. A few multinucleated giant cells with acidophilic cytoplasm were present and strap-like cells were only seen at the periphery of the sections examined. Striations were demonstrable in some strap cells and pseudorosettes were found in a few places particularly among sheets of undifferentiated round cells (Fig. 3), an appearance not unlike that seen in retinoblastoma or olfactory neuroblastoma. The picture was consistent with a rhabdomyosarcoma.

*Case III*

The patient was a 12-year-old male, who presented with a history of a rapidly growing mass in his throat for 1 month. He was admitted to another hospital because of acute respiratory difficulty, when an emergency tracheostomy was performed. A portion of the tumour, attached to the soft palate near the upper pole of the left tonsil, was later removed. Pathological examination was not carried out on this tumour. The tracheostomy cannula was *in situ* when admitted to University College Hospital, Ibadan. On examination, he was found to have a papilliferous growth in the region of the soft palate; this was removed and its base cauterized. One month later, the palatal tumour had recurred and metastases which were present in the cervical nodes continued to increase in size. They were surgically removed, along with the carotid sheath and sternomastoid muscle which were also involved.

*Pathology.*—Grossly, there were several pieces of haemorrhagic, tumour masses, muscle and a few lymph nodes. Histological examination of the tumour revealed a pleomorphic, cellular tumour with a preponderance of spindle shaped cells. There were a few large multinucleated oval cells with abundance of eosinophilic cytoplasm. In some areas the tumour was myxomatous. Striations were not demonstrable in several sections examined. Intracytoplasmic myofibrils, however, were seen in a few giant cells in the tumour on electron microscopy (Fig. 6). The lymph nodes were infiltrated by metastatic tumour. The appearance was consistent with a " rhabdomyosarcoma - type botryoides " of uvula and palate (Vieta *et al.*, 1962).

*Case IV*

The patient was a 21-year-old man who complained of 6 months' left sided nasal obstruction, toothache, left proptosis, and a mass in the left side of the neck which had appeared a few weeks before admission. On examination, he had a large, firm, smooth swelling on the left side of his face, marked proptosis of the left eye and small tumour masses beneath the upper eyelid (Fig. 2). The left nasal cavity was completely occluded by a pinkish lobulated tumour which bled easily. There were enlarged, firm, freely mobile cervical glands in the left submandibular region which were neither painful nor tender. The left proptosed eye had apparently been blind for 2 months. Radiography of the face revealed considerable bone destruction of the maxilla and part of the orbital walls. Biopsy of the tumour was obtained through a Caldwell-Luc approach. No attempt was made to define a capsule but a plane of cleavage was identified between the mucosa of the buccal sulcus and the tumour mass. Nitrogen mustard was given by the intra-aortic route and within 2 weeks, very marked reduction in tumour size was observed. Subsequently, the tumour grew larger and the left jugulo-digastric groups of lymph nodes were enlarged.

*Pathology.*—The surgical specimens received consisted of a 49 g. mass of irregular, whitish, firm tumour in several pieces and a 4 g. mass of cervical lymph nodes which were whitish and soft to touch. Histologically, the first biopsy specimen was composed of sheets of round cells with hyperchromatic nuclei and

---

EXPLANATION OF PLATES

FIG. 1.—Rhabdomyosarcoma involving the left side of face in a 1½ year old boy (Case 11). Note the oedema of the left eyelids.

FIG. 2.—Rhabdomyosarcoma of the left maxillary antrum with proptosis and exophthalmos of left eye. Note the prominence of the zygomatic arch and the thickening of the left ala nasae (Case IV).

FIG. 3.—Histological appearance of rhabdomyosarcoma in Case II. Arrows point to pseudorosettes. P.T.A.H. × 500.

FIG. 4.—Numerous large eosinophilic tumour cells metastatic to lymph node. Note the globular shape of cells. Haematoxylin & Eosin × 105.

FIG. 5.—Electron micrograph of a myofibre which is multinucleated. There are several free R.N.P. particles. N = Nucleus, n = nucleolus. Lead citrate × 4,300.

FIG. 6.—Myofibrils with Z bands in cytoplasm of myofibre. Note the presence of free myofilaments and R.N.P particles. Z = Z band. M = myofibril. Lead citrate × 32,500.

1

2

3

4

5

6

Williams, Martinson and Alli.

a few multinucleated giant cells with abundance of acidophilic cytoplasm. The second biopsy specimen revealed a highly pleomorphic cellular tumour with a preponderance of round cells, large spider cells, strap-like cells and globular shaped giant cells arranged in an alveolar pattern. The lymph nodes were almost completely replaced by sheets of large oval cells with abundance of eosinophilic cytoplasm (Fig. 4). Striations were demonstrable in the strap-like and giant cells, and myofibrils were also seen in the cytoplasm of giant cells on electron microscopy. The histological picture was one of an alveolar type of rhabdomyosarcoma arising in the maxillary antrum.

*Case V*

The patient was a 20-year-old woman, 22 weeks pregnant, who presented with a painful, swollen right eye. She claimed that 2 weeks previously, she suffered a traumatic injury to the eye and since then the eye had become progressively swollen and painful. Clinical examination revealed panophthalmitis for which evisceration of the eye was carried out. At operation, a firm tumour mass was found in the right orbit, medial to the globe from which a biopsy specimen was taken. The fixed tumour mass, 1 inch in diameter, was seen occupying the medial half of the orbit and had stretched and ulcerated the overlying eyelid. There was also a polypoid growth which occluded the right nasal airway completely. Radiography revealed an opaque right antrum with some bony destruction of the medial wall. Through a curved incision surrounding the medial half of the orbit, the greater portion of the tumour was seen to be arising in the ethmoid sinus and right nasal cavity, except where it had ulcerated and become adherent to the skin of the eyelids. No definite capsule was defined and the tumour was removed completely. Bleeding was moderate, the antrum and frontal sinuses were filled with pus but there was no visible tumour. Although the floor of the orbit had been destroyed no tumour tissue was visible. The resulting cavities were packed and postoperative recovery was uneventful.

*Pathology.*—The specimens consisted of a 1 g. biopsy specimen and a 62 g. mass of irregular greyish-yellow tumour. Histologically, both specimens showed a cellular, pleomorphic tumour with multinucleated giant cells, " tennis-racket " cells and binucleated cells with granular but acidophilic cytoplasm. Very few and doubtful striations were demonstrable on light microscopy, but myofibrils were seen in the cytoplasm of giant cells on electron microscopy. The picture was one of an embryonal rhabdomyosarcoma arising in the ethmoid sinus.

### DISCUSSION

In the field of otolaryngology, rhabdomyosarcomas are becoming more frequently recognised. In our series, the relative frequency of this type of tumour to other types of tumours of the upper respiratory tract is high—about 3 %. Out of the 5 patients presented here, 3 were males and 2 were females. The origin and primary location of tumours in the upper respiratory tract frequently present difficulties, particularly when two neighbouring organs are involved simultaneously. The tumours involved the nasal cavities in Cases I and II, soft palate in Case III, maxillary antrum in Case IV and the ethmoid sinus in Case V. The tumours in Cases IV and V arose in the paranasal sinuses but their origin is not clear. It has been suggested that these tumours probably arise from misplaced embryonic muscle cells (McCuaig, 1952). The tumour in Case III originated in the muscles of the soft palate and is similar to the botryoid type of rhabdomyosarcoma of the uvula and soft palate (Cappell and Montgomery, 1937; Vieta *et al.*, 1962). In Cases I and II, the tumours involved the nasal cavities and structures of the face including facial muscles. The location of the tumour in Case I duplicated that reported by Farra (1959) and it could be argued that the origin of this tumour was from the facial musculature. In view of the involvement of the nasal cavity, it is probably permissible to include it in this paper. Although there was diffuse involvement of the face in Case II, the tumour appeared to have originated in the nasal cavity.

There were metastatic deposits in the cervical glands in 2 cases (III and IV). The spread of the tumour to lymph nodes is consistent with rhabdomyosarcoma in general.

Until recently, many cases of rhabdomysarcoma were not diagnosed in the absence of cross striations. Stout (1946) described the various appearances of tumours arising from skeletal muscles despite the absence of demonstrable cross striations. In 2 of our cases, (III and V), cross striations were not demonstrable by light microscopy but these were studied under the electron microscope which revealed the presence of myofibrils. Of the remaining 3 cases which showed cross striations, another (Case IV) was also studied under the electron microscope for comparative purposes. The failure to demonstrate cross striations convincingly in 2 of our cases may be attributed to primary fixation in formol saline but when they were post-fixed in Zenker's solution, striations were still not evident.

There has been controversy about the demonstration of myofibrils as a prerequisite for the diagnosis of rhabdomyosarcoma. There is some evidence that while the presence of cross striations may clarify the nature of this tumour, it is not an invariable finding. Evidence is forthcoming from the present study where myofibrils were seen only on electron microscopy but not with the usual histochemical methods on conventional light microscopy.

The fine structure of the tumour cells was found to be similar in the three tumours examined. For this paper, the term " myoblast " will be used to connote the mononucleated cell destined to form a muscle cell and multinucleated cells will be referred to as " myofibres ". The presence or absence of myofilaments is not included in the characterization of cell types since myofibrillar proteins have been detected in mononucleated cells (Holtzer, Marshall and Finck, 1957).

Cross sections of the tumour cells viewed at low magnification with the electron microscope revealed myoblasts and myofibres in varying proportions, separated from each other by fibroblastic type of cells and bundles of collagen fibrils. The majority of the myofibres were binucleated and there were several indentations of their nuclear membranes. Some myofibres contained up to six nuclei each, with their cytoplasm containing varying amounts of free R.N.P. particles (Fig. 5) and myofibrils of varying length, diameter and number (Fig. 6). The myofibrils tended to be arranged in a haphazard fashion and were frequently found situated at the periphery of the cell. Free actin or myosin filaments within the cytoplasm of the myofibres were not uncommon. Although the myofibrils were frequently seen beneath the sarcolemma, this was not invariable. A few myofibrils were seen deep within the cell. Two types of free myofilaments were observed. The free thick filaments measured 170–180 Å, and the thin measured 70–80 Å, in diameter. Z bands were seen amongst some myofibrils as densely staining, amorphous bodies (Fig. 6), but other bands were not seen. This finding tends to support the hypothesis that Z bands first appear in a developing muscle and the myofilaments become secondarily attached (Wainrach and Sotelo, 1961). There is a striking paucity of sarcoplasmic reticulum in the myofibres—but this may be attributable to the degree of preservation of the materials examined.

Myoblasts, on the other hand, were smaller than myofibres and their cytoplasm contained free R.N.P. particles but devoid of endoplasmic reticulum and cytoplasmic myofilaments. The nuclear-cytoplasmic ratio was abnormal in the myoblasts because the nuclei were relatively large and in some cells, only a small amount of cytoplasm was evident.

The ultrastructural features of myogenic cells found in the three tumours examined are similar to those of embryonic chick skeletal muscle (Fischman, 1967). These findings confirm the light microscopic diagnoses of rhabdomyosarcoma in the tumours with and without demonstrable striations.

Two tumours can be classified as embryonal type of rhabdomyosarcoma (Cases I and V), one as an alveolar type (Case IV), one as a botryoid type (Case III) and one as a pleomorphic type with pseudorosettes (Case II). The demonstration of myofibrils in the tumour from Case II is consistent with the diagnosis of rhabdomyosarcoma but the predominant cell type is a round cell. In view of the presence of pseudorosettes in this tumour, the differential diagnoses considered included olfactory neuroblastoma and retinoblastoma but in neither of these are myofibrils demonstrable. We feel that the presence of pseudorosettes, observed in Case II, is unique in a rhabdomyoblastic tumour. The presence of myxomatous and fibrosarcomatous looking areas in some of the other tumours illustrates the diversity of histological appearances already described (Stout, 1946). Studies of the ultrastructure of this tumour may be rewarding particularly in cases where striations are not demonstrable by the usual techniques.

In tropical Africa, rhabdomyosarcomas affecting the paranasal sinuses or the nasofacial areas may be clinically indistinguishable from Burkitt's lymphoma, rhinophycomycosis or mucocoele of the paranasal sinuses. Distinction between these entities is important because cytotoxic therapy is of limited value in rhabdomyosarcoma but of considerable value in Burkitt's lymphoma. The prognosis in the cases of rhabdomyosarcoma is remarkably poor particularly as the available means of therapy at this institution are surgery and chemotherapy. Furthermore, many of our patients come for treatment at a late stage when complete surgical removal is not feasible. In instances, where this is possible, early recurrence and metastases are common. Chemotherapy with nitrogen mustard, actinomycin D and melphalan have been tried but without significant effects.

### SUMMARY

Rhabdomyoblastic tumours arising in and around the nasal cavity, palate and paranasal sinuses in Ibadan, Nigeria, are reported. The clinicopathological features of 5 cases are presented with the hope of drawing attention to the occurrence of the tumour at these sites and also to reaffirm the view held by some workers that these tumours are not as rare as they are generally thought to be. The diagnoses of two tumours were established from their ultrastructure. Myofibrils and free myofilaments were seen in the cytoplasm of tumour cells with similar characteristics to the embryonic chick skeletal muscle.

The authors wish to thank Dr. J. H. Yardley and Professor Edington for their help. The technical assistance of Mrs. Gertrude Brown is acknowledged, and we are indebted to Mr. F. Speed, Mr. P. Lund and Mr. M. Friedman for the photographs. One of the authors (A.O.W.) was a recipient of the Commonwealth Exchange Fellowship at Johns Hopkins Hospital, Baltimore, U.S.A., where part of this work was carried out.

### REFERENCES

ALLEN, G. W.—(1960) *A.M.A., Archs Otolar.*, **72**, 477.
BENENATI, U.—(1903) *Virchows Arch. path. Anat. Physiol.*, **171**, 418.
CAPPELL, D. F. AND MONTGOMERY, G. L.—(1937) *J. Path. Bact.*, **44**, 517.
COOPER, K. G.—(1934) *Archs Otolar.*, **20**, 329.
FARRA, C.—(1959) *Archs Otolar.*, **70**, 500.
FISCHMAN, D. A.—(1967) *J. Cell Biol.*, **32**, 557.
HOLTZER, H., MARSHALL, J. M. AND FINCK, H.—(1957) *J. biophys. biochem. Cytol.*, **3**, 705.
MCCUAIG, D. R.—(1952) *Ann. Otol. Rhinol. Lar.*, **61**, 144.
REITTER, G. S.—(1921) *J. Am. med. Ass.*, **76**, 22.
RINGERTZ, N.—(1938) *Acta otolar.*, Suppl. 27.
SHIRASAWA, K. AND TAGUCHI, H.—(1963) *Jap. J. clin. Path.*, **11**, 141.
STOUT, A. P.—(1946) *Ann. Surg.*, **123**, 447.
VIETA, L. J., GURAIEB, S. R. AND OBREGON, M. A.—(1962) *A.M.A. Archs Otolar.*, **75**, 248.
WAINRACH, S. AND SOTELO, J. R.—(1961) *Z. Zellforsch. mikrosk. Anat.*, **55**, 622.